# 3D Bioprinted Chondrogenic Gelatin Methacrylate-Poly(ethylene glycol) Diacrylate Composite Scaffolds for Intervertebral Disc Restoration

**DOI:** 10.1088/2631-7990/ad878e

**Published:** 2024-11-19

**Authors:** Maria D. Astudillo Potes, Maryam Tilton, Indranath Mitra, Xifeng Liu, Babak Dashtdar, Emily T. Camilleri, Benjamin D. Elder, Lichun Lu

**Affiliations:** 1 Mayo Clinic Alix School of Medicine, Rochester, Minnesota, USA; 2 Mayo Clinic Graduate School of Biomedical Sciences, Rochester, Minnesota, USA; 3 Department of Physiology and Biomedical Engineering, Mayo Clinic, Rochester, Minnesota, USA; 4 Department of Orthopedic Surgery, Mayo Clinic, Rochester, Minnesota, USA; 5 Department of Neurological Surgery, Mayo Clinic, Rochester, Minnesota, USA; 6 Walker Department of Mechanical Engineering, The University of Texas at Austin, Austin, Texas, USA

**Keywords:** intervertebral disc regeneration, 3D bioprinting, gelatin-based hydrogel, gelatin-based hydrogels, mesenchymal stem cell spheroids, tissue engineering, visible-light based digital light processing 3D bioprinting

## Abstract

Degenerative spine pathologies, including intervertebral disc (IVD) degeneration, present a significant healthcare challenge due to their association with chronic pain and disability. This study explores an innovative approach to IVD regeneration utilizing 3D bioprinting technology, specifically visible light-based digital light processing (VL-DLP), to fabricate tissue scaffolds that closely mimic the native architecture of the IVD. Utilizing a hybrid bioink composed of gelatin methacrylate (GelMA) and poly (ethylene glycol) diacrylate (PEGDA) at a 10% concentration, we achieved enhanced printing fidelity and mechanical properties suitable for load-bearing applications such as the IVD.

Preconditioning rat bone marrow-derived mesenchymal stem cell (rBMSC) spheroids with chondrogenic media before incorporating them into the GelMA-PEGDA scaffold further promoted the regenerative capabilities of this system. Our findings demonstrate that this bioprinted scaffold not only supports cell viability and integration but also contributes to the restoration of disc height in a rat caudal disc model without inducing adverse inflammatory responses. The study underscores the potential of combining advanced bioprinting techniques and cell preconditioning strategies to develop effective treatments for IVD degeneration and other musculoskeletal disorders, highlighting the need for further research into the dynamic interplay between cellular migration and the hydrogel matrix.

## 1. Introduction

Degenerative spine pathology involves a range of conditions that result from the wear and tear of spinal structures as well as aging leading to chronic pain and disability. Degeneration of the intervertebral disc (IVD) is a significant contributor to chronic back pain and instability, presenting a global healthcare challenge [1]. There are multiple nonoperative interventions that may help alleviate the pain associated with IVD degeneration, and when instability is present, fusion surgery is commonly performed, though this also eliminates the normal motion at this level. However, no current approaches involve restoring the native structure and functionalities of the degenerated discs. Recently, with the help of regenerative medicine strategies, the treatment landscape for IVD degeneration has significantly evolved [2–3].

Cell-based treatment approaches to regenerate functionally impaired tissue has been a promising avenue of regenerative medicine [4–5] and can restore lost function of the IVD due to degeneration on a cellular level. However, pathologies such as IVD degeneration are also associated with losing disc height due to changes in the cartilaginous and proteoglycan matrix components of the disc leading to stiffening of the matrix and eventually nerve impingement resulting in back pain and radiculopathy [6–8]. Therefore, multifactorial problems like IVD degeneration require multidisciplinary approaches especially through the combination of regenerative biomaterial and cell-based strategies such as 3D bioprinting. In this evolving landscape, 3D bioprinting has emerged as a revolutionary technology, offering the potential to fabricate tissue scaffolds that closely mimic the native architecture and functionality of the IVD [9–13].

Cell-based therapies, particularly involving 3D aggregates of cells called spheroids, offer a microenvironment that closely mimics the natural cellular milieu, promoting enhanced cell-cell interactions [14–15]. This capability is important in the context of IVD regeneration, addressing the need for a biomimetic approach due to the spatial and mechanical complexities inherent in IVD tissue. Although mesenchymal stem cell (MSC) and cartilage-based spheroid configurations have been shown to enhance IVD regeneration [16–17], the full potential of chondrogenic spheroids remains to be thoroughly explored. Notably, the pre-treatment of spheroids with chondrogenic media preconditions the cells, enhancing their chondrogenic potential without promoting full differentiation into typical hyaline cartilage chondrocytes, and potentially ensuring a more robust and effective regeneration of the IVD. This approach leverages the enhanced survival, functionality post-transplantation, and extracellular matrix (ECM) synthesis capabilities of MSCs within spheroids, as opposed to single-cell MSC configurations, alongside their augmented anti-inflammatory and proangiogenic properties, which are vital for successful tissue repair and regeneration [18–21, 16–17]. In this study, we utilized bone marrow derived MSC spheroids instead of single-layer MSCs to provide a 3D microenvironment that closely mimics native tissue, promoting cell-cell interactions and ECM secretion, which are critical for IVD repair. Spheroids exhibit enhanced paracrine signaling and offer protection from harsh conditions, improving their therapeutic efficacy in the challenging microenvironment of the IVD [17, 22–23]. Additionally, delivering MSCs in spheroid form allows for the encapsulation of large quantities of cells within the limited caudal space, facilitating the delivery of a higher cell dose. Our intention is that the cells from the spheroids will migrate outwards, further contributing to tissue regeneration through their sustained ECM production and enhanced migratory capabilities [16–17].

Recently, the use of 3D printing to fabricate tissue-regenerative scaffolds has seen significant advancements through visible light-based digital light processing (VL-DLP) techniques to crosslink polymer bioinks through the exposure to visible light [24–26]. Compared to extrusion-based techniques and other bioprinting methods, VL-DLP provides higher resolution and precision, enabling the creation of scaffolds with intricate details and complex geometries that can more closely mimic the native architecture of IVD tissue [25]. This high resolution is essential for ensuring the structural fidelity and functionality of the bioprinted scaffolds. Moreover, VL-DLP enhances cell viability by minimizing thermal and mechanical stress during the printing process, compared to extrusion-based techniques that can expose cells to detrimental shear stress and elevated temperatures [26]. Additionally, the rapid layer-by-layer fabrication of VL-DLP significantly reduces overall printing time, making it more efficient for 3D bioprinting, which is advantageous for scaling up production and progressing towards clinical applications [25].

Our prior work harnessed VL-DLP technology with hybrid gelatin methacrylate (GelMA) and poly (ethylene glycol) diacrylate (PEGDA) bioinks to create scaffolds with improved mechanical properties and enhanced cell-material interaction for tissue regeneration [24]. GelMA, a photocrosslinkable biopolymer extensively investigated in existing literature [27–29], emerges as a standout choice due to its exceptional biocompatibility, biodegradability, and excellent ability to promote cell adhesion and proliferation. Nonetheless, the inherent lack of high-fidelity printing, especially at low concentrations, and relatively low mechanical properties of GelMA have limited its application in load bearing sites such as IVDs unless tailored modifications or synergistic combinations with other materials are employed to augment its mechanical attributes. In our previous work, PEGDA, a synthetic, hydrophilic, and biocompatible polymer was combined with GelMA at 10% (w/v) concentration that showed enhanced printing resolution and fidelity while also improving the biomechanical properties of the printed scaffolds [24]. This bioink formulation was selected due to its favorable properties such as biocompatibility, tunable mechanical strength, and excellent printability. GelMA promotes cell adhesion and differentiation, while the addition of PEGDA enhances mechanical properties and printability without compromising biocompatibility. This combination creates a supportive environment for cell encapsulation, delivery, and viability, which is crucial for the regeneration of IVD tissue.

**Figure 1.**
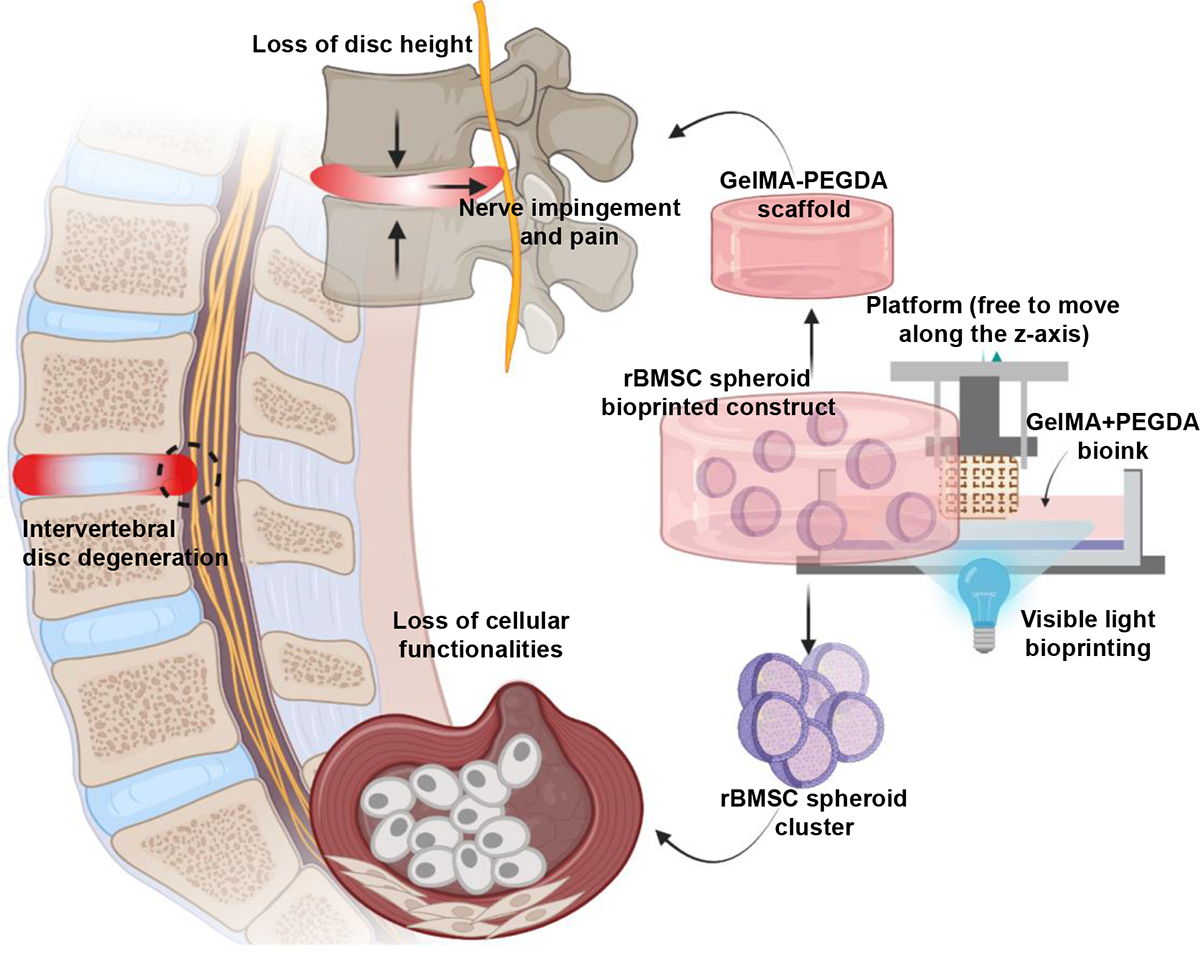
Regenerative treatment of IVD degeneration using 3D DLP bioprinted rBMSC spheroid scaffolds. The schematic highlights the strategy to restore disc height and cellular functionality by leveraging the enhanced rBMSC cell-cell and cell-matrix interactions facilitated by the biocompatible and mechanically supportive GelMA-PEGDA scaffold.

Bioprinting with the incorporation of chondrogenic spheroids in GelMA-PEGDA bioink can augment the potential of this technology in restoring the lost functionality and disc height due to IVD degeneration. Building upon our previous work, which established an optimized bioink formulation and 3D printing process, our current study delves into the regenerative capabilities of bioprinted scaffolds in IVD repair. Specifically, we investigated the effects of pre-treating rat bone marrow derived (rBM) MSC spheroids with chondrogenic media for 24 hours (short term) on subsequent IVD regeneration in a rat caudal disc model. By employing VL-DLP for the bioprinting process of these preconditioned spheroids within a 10% GelMA-10% PEGDA (GP10) bioink formulation, we aim to shed light on the synergistic potential of advanced bioprinting techniques and cellular preconditioning in fabricating bioengineered scaffolds for comprehensive IVD repair (Figure 1). Lastly, this study not only contributes to the evolving landscape of IVD tissue engineering but also sets a precedent for the use of preconditioned spheroids in 3D bioprinted scaffolds, potentially paving the way for novel regenerative therapies for other musculoskeletal disorders.

## 2. Results

### 2.1. Spheroid Formation and Hydrogel Characterization

**Figure 2.**
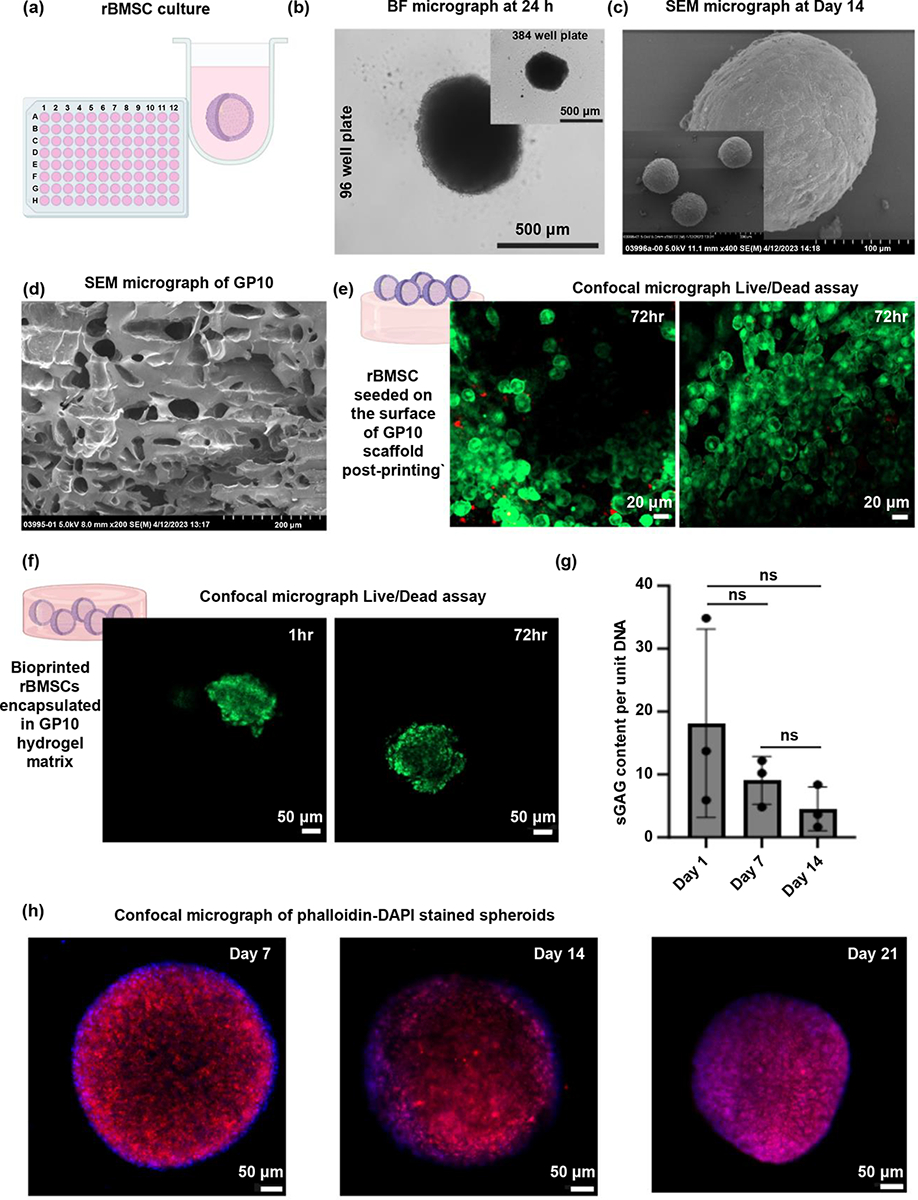
In vitro characterization of rBMSC spheroid culture. (a) Formation of rBMSC spheroids in 96-well plate, showcasing a graphical representation of a single well-formed spheroid floating within U-shaped well. (b) Bright field micrograph of a 24-hour spheroid culture confirming their formation and structural integrity within the well in both 96-well and 384-well plates. (c) SEM imaging of spheroids over a 14-day period in chondrogenic culture media indicating robust spheroid formation and cohesive structure at lower and higher magnifications. (d) SEM imaging of GP10 hydrogel highlighting its inherent microporous structure underlining its potential for cellular integration. (e) Representative high biocompatibility and cellular migration of spheroids seeded on top of the GP10 hydrogel scaffold after 72 hours of culture using live/dead assay. (f) Spheroids encapsulated within hydrogel matrix maintained viability and consistent formation at 1 hour and for 72 hours post-bioprinting. (g) sGAG content per unit DNA analysis for 14 days and (h) phalloidin staining up to day 21 indicate cellular activity and ECM dynamics in long-term culture of the bioprinted rBMSC spheroids within GP10. All in vitro experiments used n=3 per group. Ns= non-significant findings with p-value set at 0.05.

Spheroids were efficiently generated using 96-well and 384-well plates, with their formation verified through microscopic imaging at 24 hours post-culture (Figure 2(b)). Over a 14-day culture period in chondrogenic media, the spheroids maintained a cohesive and well-defined morphology (Figure 2(c)), suggesting a stable microenvironment conducive to cellular activities. Further, post hoc mechanical characterization of the spheroids in basal culture, using optical fiber-based interferometry nanoindentation at days 3 and 10 post-culture, revealed no significant difference in their Young’s modulus. These findings indicate that spheroids maintain stable mechanical properties in the early stage of culture (Supplemental Figure 3), highlighting the resilience and potential of spheroids for use in tissue engineering.

Our previous work revealed that GP10 hydrogels significantly advanced scaffold properties for scalable tissue engineering, highlighted by reduced swelling, improved photocrosslinking with gel fractions over 85%, controlled biodegradation, with more than 60% of the structure retained after 10 weeks in enzymatic conditions, and enhanced compressive modulus (approximately 75 kPa) and strength (45 kPa) compared to GelMA alone [24]. The optimized GP10 composition provides a balance between elasticity, shape-recovery, and rigidity, mimicking the natural ECM of the disc tissue while ensuring a more adequate mechanical support. Additionally, the GP10 hydrogel’s surface free energy values, around 40.12 mN·m^−1^ for 10% PEGDA, compared to the GelMA only control’s 49.93 mN·m^−1^, ensured maintained hydrophilicity, which is key for cell adhesion and spreading [24]. Scanning electron microscopy (SEM) analysis of GP10 hydrogel confirmed a well-ordered, interconnected microporous structure, essential for cell infiltration and nutrient diffusion (Figure 2(d)). The optimized GP10 composition provides a balance between flexibility and rigidity, mimicking the natural ECM of the disc tissue while ensuring a more adequate mechanical support.

### 2.2. Cell Viability and Integration within Hydrogel

Direct interaction between the spheroids, culture media, and the GP10 hydrogel demonstrated remarkable biocompatibility and cellular migration as seen by the live cells spreading on top of the hydrogel at 72 hours post-seeding (Figure 2(e)), an essential factor for subsequent in vivo applications. Spheroids encapsulated within the hydrogel matrix exhibited sustained viability, both immediately after bioprinting (1 hour) and at 72 hours, indicating successful encapsulaiton within the biomaterial (Figure 2(f)). The maintenance of spheroid structure within the bioprinted matrix after 72 hours underscores the potential of using GP10 as a carrier for localized delivery of spheroids with well-defined shape (Figure 2(f)).

### 2.3. Long-term Culture, ECM Production, and Degradation Dynamics

Chondrogenic induction of the bioprinted spheroids over a 21-day period revealed sustained cellular activity, with phalloidin staining confirming the presence of actin filaments within the hydrogel up to day 21 (Figure 2(h)). However, a noted trend of decreasing sulfated glycosaminoglycans (sGAG) content per unit DNA by day 14, though non-significant (p < 0.05), suggests alterations in ECM production potentially due to cell migration and cell-laden hydrogel degradation (Figure 2(g)). This observation emphasizes the importance of balancing cellular migration and hydrogel degradation with tissue formation rates, particularly in the context of IVD regeneration where scaffold integrity and mechanical support are critical.

### 2.4. In Vivo Implantation and Tissue Response

Caudal disc levels C5–9 were selected for their uniform height [30], and the four groups (intact, GP10 only, rat bone marrow-derived mesenchymal stem cells (rBMSC)-GP10, and empty control) were randomly allocated to these discs to minimize level-specific effects (Figure 4(a)). Hydrogel scaffolds with an average of 15 spheroids per scaffold were selected for implantation to ensure uniformity in the in vivo implantation results. Figure 4(b) depicts an example of the surgical caudal sites with a high-density scaffold (greater than 20 spheroids) of the same dimensions as the implanted hydrogel scaffolds. Notably, the variability in spheroid density per scaffold highlighted the challenges in standardizing bioprinted scaffolds using available VL-DLP systems (Lumen X), a crucial consideration for clinical translation. (Supplemental Figure 1).

**Figure 3.**
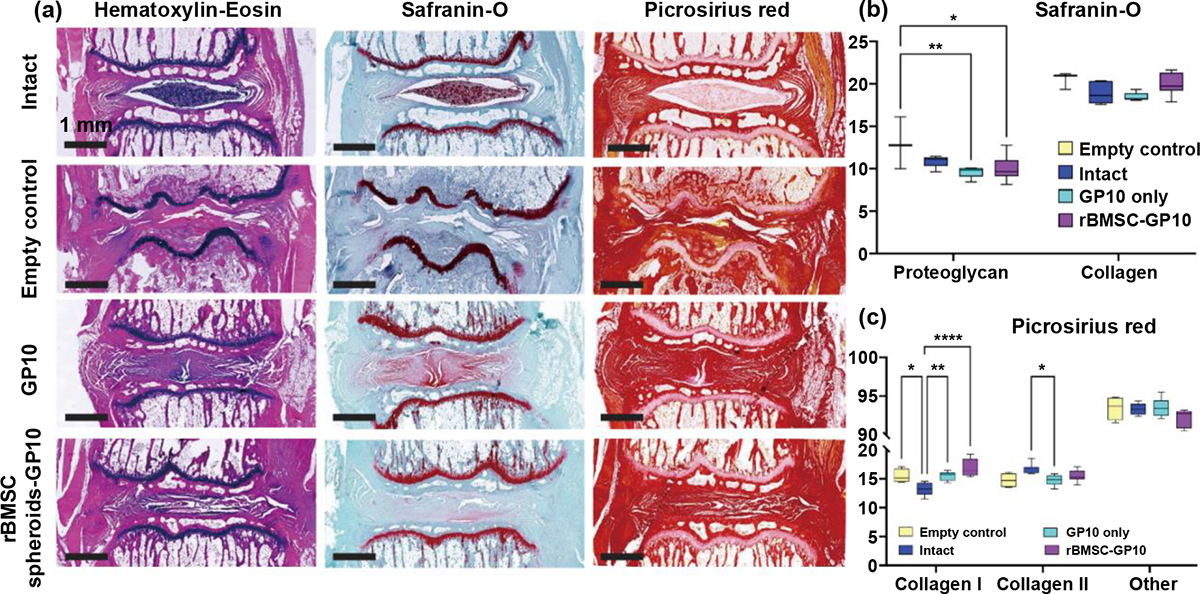
Representative histological and quantitative histomorphometry. (a) Histological evaluation of tissue response post-implantation at 4 weeks as seen with Hematoxylin and Eosin (H&E) for cellular and structural composition of discs, Safranin-O counterstained with Fast Green (SafO), and Picrosirius Red (PR) staining of the four groups (intact, GP10 only, rBMSC-GP10, and empty control). (b) Quantification of SafO proteoglycan vs. collagen content within the discs. (c) Quantification of PR collagen type distribution with notable observations regarding type I collagen content among the different groups. Ns= non-significant findings with p-value set at 0.05, **** = p < 0.000 1, *** = p < 0.001, ** = p < 0.01, and *= p < 0.05.

Histological analysis using Hematoxylin and Eosin (H&E), Safranin-O/Fast Green (SafO), and Picrosirius red (PR) staining was performed to assess proteoglycan and collagen content within the four groups (Figure 3(a)). SafO staining indicated a preservation of proteoglycan and overall collagen content between the intact and other groups (Figure 3(b)). While proteoglycan content appeared to significantly decrease between the empty control and treatment groups: GP10 only (mean difference = 3.34, 95% CI [0.75, 5.93], p < 0.01) and rBMSC-GP10 (mean difference = 2.97, 95% CI [0.57, 5.37], p < 0.05), overall collagen content did not change. However, PR staining offered a nuanced understanding of the collagen composition, including type I and type II collagens and distribution within the discs (Figure 3(c)). Type I collagen was significantly lower in the intact group compared to the other groups: rBMSC-GP10 (mean difference = − 3.59, 95% CI [−5.21, −1.97], p < 0.000 1), GP10 only (mean difference = −2.31, 95% CI [−4.04, −0.58], p < 0.01), and empty control (mean difference = 2.21, 95% CI [0.28, 4.15], p < 0.05) consistent with normal IVD phenotype. Yet, type II collagen did not appear to significantly vary within the groups, except for between the intact and GP10 hydrogel only groups (mean difference = 1.79, 95% CI [0.06, 3.52], p < 0.05).

### 2.5 Micro-CT Caudal Disc Height Quantification

**Figure 4.**
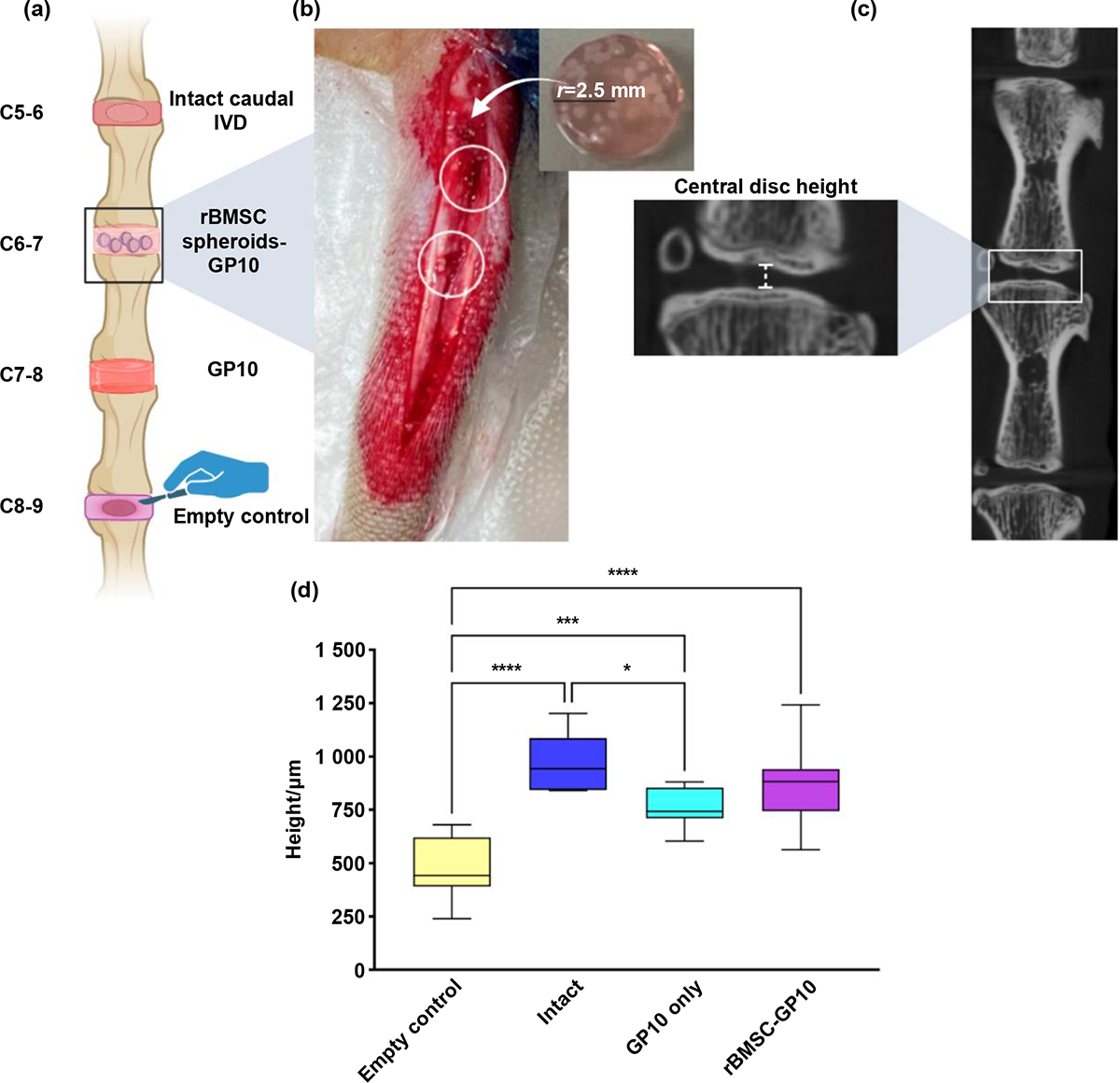
In vivo implantation and morphological assessment. (a) Illustration of the four groups, which were randomly allocated across caudal disc levels C5–9 for each rat to ensure uniformity and minimize level-specific effects. (b) Example of surgical implantation of high-density spheroid hydrogel scaffold into a caudal disc highlighting the procedure and dimensions of implanted scaffold. (c) Representative micro-CT images and (d) measurements of central disc height across the groups with a focus on the restoration of disc height in the rBMSC spheroids-GP10 group to levels comparable to the intact group. Ns= non-significant findings with p-value set at 0.05, **** = p < 0.000 1, *** = p < 0.001, ** = p < 0.01, and *= p < 0.05.

The micro-computed tomography (CT)-based quantification of central disc height further confirmed our animal model (as seen with a significant decrease in disc height after 4 weeks in the empty control group compared to the intact group: mean difference = −494.30, 95% CI [−662.60, −326.0], p < 0.000 1) and substantiated the scaffold’s efficacy in preserving disc height. Particularly, disc height was significantly lower in the GP10 only group compared to the intact group (mean difference = 204.7, 95% CI [36.42, 373.0], p < 0.05), albeit not to the extent observed in the empty control group, while effectively maintained in the rBMSC spheroids-GP10 group comparable to native intact levels (mean difference = 108.50, 95% CI [−59.73, 276.80], p = 0.32) (Figure 4(d)). This highlights the pivotal role of spheroid integration in restoring disc height to its physiological range. These findings, coupled with the absence of inflammatory markers or responses, including necrosis and neoplasia in major rat organs (Supplemental Figure 2), reinforce the biocompatibility of our bioink formulation, making it a promising candidate for IVD regeneration.

## 3. Discussion

Intervertebral disc degeneration involves numerous failures at the cellular, biochemical, and mechanical levels. This degenerative process is influenced by a complex interplay of age, nutritional, and mechanical factors [31], leading to alterations in the cellular structure and ECM of the disc. The imbalance between matrix synthesis and degradation contributes to its progressive degeneration, exacerbated by the limited capacity for self-repair, thus affecting spinal biomechanics. On a cellular level, there’s a decline in the overall chondrocyte density within the disc and an acceleration in the aging of these chondrocytic cells, leading to their decreased functionality due to necrosis and apoptosis, impacting aging biology and ECM production [32–33]. The changes in chondrocyte numbers and their function result in modifications of the cartilaginous and proteoglycan matrix components. Decreased production by aging disc cells and increased breakdown by proteolytic enzymes cause proteoglycan loss [6–8], resulting in disc dehydration and collapse. Similarly, the collagenous matrix undergoes changes, leading to fibrosis, reduced flexibility, and altered load distribution contributing to the loss of disc height.

This study represents a continued effort towards addressing these pressing clinical needs for effective and innovative treatments for IVD degeneration. We have developed a 3D bioprinting-based two-pronged approach where rBMSC spheroids (Figure 2(b)) can restore tissue functionality by mimicking the natural milieu of the IVD environment and the GP10 fabricated microporous scaffold (Figure 2(d) and (f)) that encapsulates the spheroids not only provides a suitable matrix for cell-cell interactions but also serves as a porous 3D structure implanted to restore the disc height and potentially integrates with the endplates to stabilize at the implant location.

Our findings pertaining to cell viability within the hydrogel and restoration of disc height are particularly compelling (Figures 2(e), (f), and 4(d)). The observed biocompatibility and cellular migration within the GP10 hydrogel matrix underscore its potential as an effective carrier for spheroids, furthering prior research that observed excellent compatibility of this particular bioink formulation on cell attachment and migration on the surface of 3D printed scaffolds [24]. Overall, the GP10 combination not only enhances mechanical strength compared to GelMA alone but also supports cell viability, making it an ideal candidate for IVD repair.

The application of VL-DLP bioprinting technology allows for the precise fabrication of scaffolds with tailored architecture and mechanical properties. The use of visible light to crosslink the GP10 bioink containing rBMSC spheroids to fabricate the scaffolds is crucial for the survival of the cells during the printing process. Furthermore, the optimized printing parameters such as concentration and degree of crosslinking in our prior study ensured the maintenance of cell viability post-printing for 72 h, both on the surface (Figure 2(e)) and within the matrix (Figure 2(f)), a critical factor for the subsequent in vivo efficacy of the scaffolds. Furthermore, long-term 14- and 21-days in vitro assessment revealed sustained cellular activity within the GP10 matrix alongside noticeable alterations in ECM production. Evident phalloidin presence in the rBMSC spheroids over 21 days revealed the organization of the actin cytoskeleton maintaining the 3D aggregated spherical structure of the cells (Figure 2(h)).

The lack of a significant increase in sGAG per unit of DNA observed in our study may be attributed to several factors aligned with our intended design goal of promoting cell migration through the hydrogel matrix. One plausible explanation is the anticipated loss of cells from the spheroids during subsequent media changes, impacting the overall cell count and sGAG production. Additionally, the decreasing trend in the size of spheroids from day 7 to day 21 suggests that the rBMSCs were indeed migrating locally out of the aggregate structure and into the surrounding hydrogel matrix, as intended. This migration is further corroborated by the changes in sGAG content from day 1 to day 14 when normalized over DNA content (Figure 2(g)). This observation warrants further investigation into the dynamic interplay between cellular migration and the hydrogel matrix via specific assay development, live cell staining, and image-based tracking. Lastly, achieving a balance between promoting chondrogenic differentiation and maintaining original stem cell properties needs to be further elucidated for potential effective IVD repair.

The in vivo application of the bioprinted scaffolds in a rat caudal disc model provided valuable insights supporting the efficacy of the GP10 hydrogel in IVD regeneration [34–37]. Histological staining of disc sections with H&E revealed no evidence of inflammatory response towards the implant biomaterial as well as no signs of neoplasia and necrotic damage (Figure 3(a)). Histomorphometric quantification of SafO stained sections for proteoglycans and collagen showed overall higher presence of collagen compared to proteoglycans in all groups including the empty control (evaluated for only the endplates) (Figure 3(b)). Groups GP10 and rBMSC-GP10 showed no significant difference in proteoglycan content compared to the intact IVD, likely due to the porous hydrophilic GP10 hydrogel facilitating the diffusion of these large molecules. SafO staining did not provide a nuanced assessment of the difference in collagen content between the groups evidenced from no statistical difference, likely due to a uniform blue color of overall collagen compared to the distinct red of the proteoglycans marked as classifiers in the segmentation process.

The lack of a significant difference in collagen content between the samples with and without cells can be attributed to several factors. First, the GP10 hydrogel scaffold itself may provide a baseline level of collagen deposition, supporting the formation of a basic collagen matrix even in the absence of cells. Second, while rBMSCs are capable of producing collagen, this study focused on the initial (1-month post-implantation) effects, where cells are dynamically producing various components based on the immediate needs of the regenerating tissue. Over time, the total quantities and composition of these components might evolve as the cells continue to differentiate and produce ECM components necessary for later stages of tissue development. Additionally, the sensitivity and variability of the methods used to quantify collagen might mask subtle differences between groups. Finally, the focus on chondrogenic differentiation might have led the rBMSCs to prioritize the production of other ECM components, such as glycosaminoglycans and type II collagen, rather than significantly increase the overall collagen content detectable by our assays. These factors indicate ongoing tissue regeneration and highlight the dynamic nature of ECM production at different stages of the healing process, warranting further investigation over longer study durations.

Therefore, to assess the presence of collagens, PR staining was employed and quantified for the collagens, including type I and type II (Figure 3(c)). Type I collagen is known for its structural role, providing tensile strength to tissues. However, the IVD is a complex structure where mechanical integrity is not solely dependent on the presence of a single type of collagen but a result of the intricate composition of the ECM, including proteoglycans, type II collagen, and other components. It’s plausible that the observed decrease in type I collagen in the intact group compared to the empty control might have been compensated by other matrix components such as the observed increase in type II collagen and due to the fact that the empty controls were a measure of the cartilaginous end plates which were not removed as a part of the surgical procedure. However, the GP10 and rBMSC-GP10 scaffolds showed higher type I collagen presence compared to the intact group, suggesting a synergistic action of the spheroids and the hydrogel biomaterial and a lower amount of type II collagen in GP10 group compared to the intact group.

The micro-CT analyses offered a comprehensive evaluation of the structural response to the implanted scaffolds (Figure 4(c) and (d)). We have observed restoration of disc height for GP10 and rBMSC-GP10 groups evidenced from significant difference compared to the empty control with an increasing trend towards the native disc height of the intact group. The observations regarding the preservation of disc height in the rBMSC spheroids-GP10 group, despite differences in type I collagen levels, suggests that 4-week implantation might not be sufficient for a bioprinted scaffold like the one presented in this research to provide statistically significant results towards matrix component restoration of the IVD. However, the trend observed in this study as well as some of the statistical differences present an intriguing aspect that merits further longer-term examination. Notably, our results showed a clear indication of disc height restoration taking precedence over ECM restoration due to the implantation of a 3D bioprinted scaffold. Additionally, the chondrogenic preconditioning of the spheroids before bioprinting could have played a pivotal role in promoting a more cartilage-like cellular phenotype. This phenotypic adaptation might have contributed to the maintenance of disc height by fostering a matrix composition more akin to native IVD tissue, despite the variations in type I collagen levels. Lastly, insights gained from our post-hoc analysis of spheroid stiffness underscore the importance of further investigating the integration of mechanical cues with chondrogenic induction. Such research is vital for the development of regenerative strategies that more accurately replicate the native biomechanical environment of the IVD. The promising preliminary findings in tissue response, disc height preservation, and absence of adverse inflammatory markers set the stage for further investigations into the long-term efficacy and safety of bioprinted scaffolds.

## 4. Conclusion

Our study not only sheds light on the potential of GelMA-PEGDA hydrogels and rBMSC spheroids in fabricating bioprinted scaffolds for IVD regeneration but also highlights the significance of precise bioprinting techniques that are required for a comprehensive in vitro and in vivo evaluation of IVD regenerative strategies. The precedence of disc height restoration over ECM production suggests a conducive nature of this bioprinted scaffold in relieving patients from the more immediate symptoms of IVD degeneration such as disc collapse and nerve impingement associated with back pain. The maintenance of disc height in vivo, even with altered collagen composition, suggests a complex interplay of factors contributing to the structural and functional integrity of the regenerated IVD tissue. Future studies are encouraged to build on this foundation, exploring long-term outcomes and further elucidating the mechanisms underlying the regenerative processes observed.

## 5. Experimental section

### 5.1. GP10 scaffold fabrication

GelMA (medium methacrylation, ~50%) was synthesized as per established protocols [24, 35–36]. Briefly, type A porcine skin gelatin (10% w/v) was dissolved in Dulbecco’s phosphate buffered saline (DPBS; Gibco, Carlsbad, CA) at 50 °C. Methacrylic anhydride (MA) was added dropwise at a controlled pace of 0.5 mL·min^‒1^ to the solution, maintaining the temperature, and stirred for 2 hours. Post-reaction, the mixture was diluted threefold with DPBS, dialyzed against distilled water for one week using 12‒14 kDa cutoff tubing, and lyophilized to form a porous white foam. The final product was stored at −20 °C. To prepare our hydrogel bioink, lyophilized GelMA was dissolved in sterile DPBS at 50 °C to achieve a 10% w/v concentration. Simultaneously, PEGDA (average Mn 575, Sigma-Aldrich, St. Louis, MO) at a 10% w/v concentration was mixed under sterile conditions to obtain a homogenous 10% GelMA-10% PEGDA (GP10) solution. Prior to printing, the bioink was sterilized using a 0.22 μm syringe filter and combined with the photoinitiator lithium phenyl-2, 4, 6-trimethylbenzoylphosphinate (LAP) at a 0.05% w/v concentration and the photoabsorber tartrazine at a 0.005% w/v concentration, ensuring a photocrosslinkable mixture previously optimized for VL-DLP bioprinting.

### 5.2. rBMSC spheroid culture

rBMSCs from the Sprague-Dawley strain (Catalog No. S1601-100, Gibco) were cultured in basal medium composed of low-glucose Dulbecco’s Modified Eagle’s Medium (DMEM, Sigma-Aldrich) supplemented with 10% fetal calf serum (FCS, Gibco), 2 mM L-glutamine (GlutaMAX^™^, Gibco) and 0.5% penicillin-streptomycin (Pen-Strep, Gibco) in a controlled environment (37 °C, 5% CO^2^, 95% humidity).

Using Corning spheroid microplates (96-well and 384-well, Corning, Kennebunk, ME), 50 μL of rBMSC cell suspension was added to each well to form spheroids at a concentration of 2 × 10^4^ cells per well. The initial cell seeding densities were quantified using a hemocytometer gridline to ensure accurate cell counts prior to spheroid formation. The plate was centrifuged at 1 500 rpm for 5 minutes before incubation. The mechanical properties, mainly the Young’s modulus as a measure of stiffness, of the spheroids were assessed using optical fiber-based interferometry nanoindentation (Pavone, Optics11 Life, Netherlands). These measurements were conducted on spheroids cultured in basal media at days 3 and 10 post-culture. To evaluate the impact of chondrogenic differentiation media (PromoCell, Sigma-Aldrich) on the spheroids, the spheroids were initially cultured in basal medium for 24 hours and observed under an inverted laser scanning confocal microscope (Carl Zeiss, Germany). Subsequently, the culture medium was replaced with the chondrogenic differentiation medium, and the spheroids were further incubated for an additional 24 hours to facilitate preconditioning prior to bioprinting. SEM was used to confirm shape of spheroids up to 14 days in culture (96-well plate).

### 5.3. VL-DLP of rBMSC spheroids with GP10 bioink

The GP10 bioink was prepared as previously mentioned to a volume of 1 ml for printing of hydrogel control scaffolds (GP10 only) and to a volume of 800 μl for bioprinting with spheroids (rBMSC spheroids-GP10). SEM was used to assess the microporosity of the GP10 scaffolds. The rBMSC spheroids were suspended in 200 μl of culture media to achieve a concentration of 10 million cells per ml (approximately 500 spheroids). This suspension was then gently mixed with the 800 μl of bioink for a final volume of 1 ml, ensuring uniform distribution of spheroids within the ink. The prepared GP10 bioink laden with rBMSC spheroids was gently mixed as it was loaded onto the sterile vat of the VL-based DLP system (Lumen X, Cellink, Sweden) immediately prior to bioprinting. The printing bed was heated to 60 °C and the parameters were set as follows to ensure optimal scaffold structure and cellular viability: 50% light intensity, 50 μm thickness of each printed layer, 20s exposure time per layer, and 4x initial layer exposure time. After bioprinting, cell-laden scaffolds (2.5 mm radius × 1 mm height cylinders) were carefully removed from the printing head, rinsed with sterile DPBS and placed in chondrogenic differentiation media for 24 hours in the incubator prior to implantation.

Spheroid viability was assessed using the LIVE/DEAD^®^ Cell Imaging Kit (Thermo Fisher Scientific, Waltham, MA) at 1 hr and 72 hr post-seeding (spheroids placed on top of the GP10 only hydrogel scaffold) and post-bioprinting. Total sGAG within the rBMSC spheroid laden GP10 hydrogels was quantified using the Blyscan GAG Assay (Biocolor Ltd, UK), while DNA content was measured via the CyQUANT DNA Assay (Thermo Fisher Scientific). These assays provided a reliable measure of ECM production and cell proliferation, respectively, with the ratio of sGAG to DNA offering a normalized metric for evaluating the hydrogel scaffolds’ bioactivity.

### 5.4. Rat caudal disc model

Nineteen 12-week-old male Sprague Dawley rats underwent tail surgery to injure the caudal discs as a model to study IVD regeneration. All procedures were done with the approval of our Institutional Animal Care and Use Committee (protocol A00006647-22). Based on previously established protocols [34, 37–39], a dorsal incision using a #15 blade exposed the caudal discs (C5–9), and depending on the group, discs were either left intact (positive control) or crushed effectively removing IVD tissue. To test the effect of our hydrogel control and spheroid laden scaffolds, caudal discs C5–9 were randomly stratified into the following 4 groups: intact (positive control), empty control (crushed without treatment), GP10 only (crushed followed by hydrogel scaffold implantation), and rBMSC spheroid-GP10 (crushed followed by spheroid laden scaffold implantation), ensuring adequate distribution and representation to minimize IVD level-specific effects. Spheroids per scaffold were manually counted and Z-stacking + area stitching on confocal microscope was performed to help with counting number of spheroids prior to in vivo implantation to ensure uniformity in results. Caudal disc access was facilitated by sharp and blunt dissections to ensure minimal disturbance to the surrounding structures and protection to the ventral tail artery and veins. Implantation and any necessary tendon repairs were meticulously performed under 2.5X loop magnification prior to fascia and skin closure. Euthanasia was conducted at 4 weeks post-procedure, following American Veterinary Medical Association guidelines. Three rats were excluded from analysis due to post-operative complications, leaving 16 for analysis.

### 5.5. Histology and micro-CT analysis

Rat tails were harvested 4 weeks following implantation. Skin was removed but longitudinal tendons and surrounding soft tissue were left intact to avoid disrupting the hydrogel implants during tissue processing. Subsequently, all samples were thoroughly washed with DPBS and fixed in 4% paraformaldehyde for 48‒72 hours at room temperature. High-resolution micro-CT scanning was employed to evaluate the structural integrity and spatial configuration of the caudal discs. Scans were performed using a Bruker Micro-CT Scanner (Bruker Corporation, Billerica, MA) for all 16 tail specimens. For histological evaluation, 8 rat tail samples were randomly selected, providing insights into the tissue’s response to the different treatments. Decalcification, dehydration, embedding in paraffin, sectioning at a thickness of 5 μm, SafO, PR, and H&E staining were done by the Mayo Clinic Histology Core.

#### 5.5.1 Quantitative Histomorphometry

The stained tissue sections were imaged under a digital brightfield microscope and histomorphometry analysis was carried out using Trainable Weka Segmentation in ImageJ [40–42]. The SafO stained sections were observed for and proteoglycans while the PR-stained sections were observed for types of collagens present in the regenerated tissue namely collagen I, collagen II and other. H&E-stained sections were evaluated for any visible markers of inflammatory response [43]. For SafO and PR stains, the tissue components observed were set as classifiers and quantified as the average area fraction % of each classifier calculated using Trainable Weka Segmentation in ImageJ over 10 subsequent sections around the scaffold under a high-power field (×10) around the IVD region chosen based on established region of interest [30, 44].

#### 5.5.2 Central disc height quantification

To quantitatively assess the morphological changes in the caudal IVDs post-treatment, central disc height was measured using high-resolution micro-CT scans. This measurement extended from one vertebral endplate to another, utilizing the mid-sagittal section [30]. To ensure the integrity of the data, all caudal discs visible on the scans were measured blindly. Subsequently, these measurements were accurately allocated to their respective groups for thorough analysis, effectively minimizing potential bias. This non-invasive imaging technique facilitated a precise evaluation of the IVD structure at the disc’s center, yielding crucial insights into disc integrity and delineating the extent of potential regeneration or degeneration.

### 5.6 Statistical analysis

A one-sample t-test was employed to analyze the in vitro sGAG content per unit DNA in the rBMSC spheroid-GP10 scaffolds. To assess the variance in SafO and PR staining across different groups for multiple staining components, a two-way analysis of variance (ANOVA) was utilized. Additionally, a one-way ANOVA was conducted to compare the height measurements among the four study groups, determining the significance of differences in mean heights. Post-hoc Tukey analyses were performed following ANOVA to identify specific group differences. The threshold for statistical significance was set at a p-value less than 0.05. Analyses were done using the GraphPad Prism 10.1.2 software.

## Supplementary Material

1

## List of abbreviations

IVD: intervertebral disc
VL-DLP: visible light-based digital light processing
GelMA: gelatin methacrylate (GelMA)
PEGDA: poly(ethylene glycol) diacrylate
GP10: 10% GelMA-10% PEGDA
LAP: lithium phenyl-2, 4, 6-trimethylbenzoylphosphinate
rBMSC: rat bone marrow derived mesenchymal stem cell
ECM: extracellular matrix
SEM: scanning electron microscopy
sGAG: sulfated glycosaminoglycan

## References

[1] Costăchescu B, Niculescu AG, Teleanu RI, Iliescu BF, Rădulescu M, Grumezescu AM and Dabija MG. 2022. Recent advances in managing spinal intervertebral discs degeneration. Int. J. Mol. Sci 23, 6460. 35742903 10.3390/ijms23126460 PMC9223374

[2] Soufi KH, Castillo JA, Rogdriguez FY, DeMesa CJ and Ebinu JO. 2023. Potential role for stem cell regenerative therapy as a treatment for degenerative disc disease and low back pain: a systematic review. Int. J. Mol. Sci 24, 8893. 37240236 10.3390/ijms24108893 PMC10219191

[3] Guo TW, Zhang XB, Hu YC, Lin MQ, Zhang RH, Chen XY, Yu DC, Yao X, Wang P and Zhou HY. 2022. New hope for treating intervertebral disc degeneration: microsphere-based delivery system. Front. Bioeng. Biotechnol 10, 933901. 10.3389/fbioe.2022.933901 PMC9343804 35928951

[4] Ullah I, Subbarao RB and Rho GJ. 2015. Human mesenchymal stem cells - current trends and future prospective. Biosci. Rep 35, e00191. 25797907 10.1042/BSR20150025 PMC4413017

[5] Trounson A and McDonald C. 2015. Stem cell therapies in clinical trials: progress and challenges. Cell Stem Cell 17, 11–22. 26140604 10.1016/j.stem.2015.06.007

[6] Roberts S, Caterson B, Menage J, Evans EH, Jaffray DC and Eisenstein SM. 2000. Matrix metalloproteinases and aggrecanase: their role in disorders of the human intervertebral disc. Spine (Phila Pa 1976) 25, 3005–3013. 11145811 10.1097/00007632-200012010-00007

[7] Pockert AJ, Richardson SM, Le Maitre CL, Lyon M, Deakin JA, Buttle DJ, Freemont AJ and Hoyland JA. 2009. Modified expression of the ADAMTS enzymes and tissue inhibitor of metalloproteinases 3 during human intervertebral disc degeneration. Arthritis Rheum. 60, 482–491. 19180493 10.1002/art.24291

[8] Bachmeier BE, Nerlich A, Mittermaier N, Weiler C, Lumenta C, Wuertz K and Boos N. 2009. Matrix metalloproteinase expression levels suggest distinct enzyme roles during lumbar disc herniation and degeneration. Eur. Spine J 18, 1573–1586. 19466462 10.1007/s00586-009-1031-8 PMC2899407

[9] Mandrycky C, Wang ZJ, Kim K and Kim DH. 2016. 3D bioprinting for engineering complex tissues. Biotechnol. Adv 34, 422–434. 26724184 10.1016/j.biotechadv.2015.12.011 PMC4879088

[10] Deptuła M, Zawrzykraj M, Sawicka J, Banach-Kopeć A, Tylingo R and Pikuła M. 2023. Application of 3D-printed hydrogels in wound healing and regenerative medicine. Biomed. Pharmacother 167, 115416. 10.1016/j.biopha.2023.115416 37683592

[11] De Pieri A, Byerley AM, Musumeci CR, Salemizadehparizi F, Vanderhorst MA and Wuertz - Kozak K. 2020. Electrospinning and 3D bioprinting for intervertebral disc tissue engineering. JOR Spine 3, e1117. 33392454 10.1002/jsp2.1117 PMC7770193

[12] Sun BB, Lian MF, Han Y, Mo XM, Jiang WB, Qiao ZG and Dai KR. 2021. A 3D-Bioprinted dual growth factor-releasing intervertebral disc scaffold induces nucleus pulposus and annulus fibrosus reconstruction. Bioact. Mater 6, 179–190. 32913927 10.1016/j.bioactmat.2020.06.022 PMC7451922

[13] Yoshida M, Turner PR and Cabral JD. 2023. Intervertebral disc tissue engineering using additive manufacturing. Gels 9, 25. 10.3390/gels9010025 PMC9857857 36661793

[14] Białkowska K, Komorowski P, Bryszewska M and Miłowska K. 2020. Spheroids as a type of three-dimensional cell cultures—examples of methods of preparation and the most important application. Int. J. Mol. Sci 21, 6225. 32872135 10.3390/ijms21176225 PMC7503223

[15] Griffin KH, Fok SW and Kent Leach J 2022. Strategies to capitalize on cell spheroid therapeutic potential for tissue repair and disease modeling. npj Regen. Med 7, 70. 36494368 10.1038/s41536-022-00266-z PMC9734656

[16] Wang YY 2022. Spheroid formation enhances the regenerative capacity of nucleus pulposus cells via regulating N-CDH and ITGβ1 interaction. Int. J. Biol. Sci 18, 3676–3696. 35813471 10.7150/ijbs.70903 PMC9254483

[17] Kasamkattil J, Gryadunova A, Martin I, Barbero A, Schären S, Krupkova O and Mehrkens A. 2022. Spheroid-based tissue engineering strategies for regeneration of the intervertebral disc. Int. J. Mol. Sci 23, 2530. 35269672 10.3390/ijms23052530 PMC8910276

[18] Bartosh TJ, Ylöstalo JH, Mohammadipoor A, Bazhanov N, Coble K, Claypool K, Lee RH, Choi H and Prockop DJ. 2010. Aggregation of human mesenchymal stromal cells (MSCs) into 3D spheroids enhances their antiinflammatory properties. Proc. Natl. Acad. Sci. USA 107, 13724–13729. 20643923 10.1073/pnas.1008117107 PMC2922230

[19] Achilli TM, Meyer J and Morgan JR. 2012. Advances in the formation, use and understanding of multi-cellular spheroids. Expert Opin. Biol. Ther 12, 1347–1360. 22784238 10.1517/14712598.2012.707181 PMC4295205

[20] Cesarz Z and Tamama K. 2016. Spheroid culture of mesenchymal stem cells. Stem Cells Int. 2016, 9176357. 10.1155/2016/9176357 PMC4663368 26649054

[21] Murphy KC, Whitehead J, Falahee PC, Zhou DJ, Simon SI and Leach JK. 2017. Multifactorial experimental design to optimize the anti-inflammatory and proangiogenic potential of mesenchymal stem cell spheroids. Stem Cells 35, 1493–1504. 28276602 10.1002/stem.2606 PMC5446296

[22] Kang Y, Na J, Karima G, Amirthalingam S, Hwang NS and Kim HD. 2024. Mesenchymal stem cell spheroids: a promising tool for vascularized tissue regeneration. Tissue Eng. Regen. Med 21, 673–693. 38578424 10.1007/s13770-024-00636-2 PMC11187036

[23] Yen BL, Hsieh CC, Hsu PJ, Chang CC, Wang LT and Yen ML. 2023. Three-dimensional spheroid culture of human mesenchymal stem cells: offering therapeutic advantages and in vitro glimpses of the in vivo state. Stem Cells Transl. Med 12, 235–244. 37184894 10.1093/stcltm/szad011 PMC10184701

[24] Tilton M, Camilleri ET, Astudillo Potes MD, Gaihre B, Liu XF, Lucien F, Elder BD and Lu LC. 2023. Visible light-induced 3D bioprinted injectable scaffold for minimally invasive tissue regeneration. Biomater. Adv 153, 213539. 10.1016/j.bioadv.2023.213539 PMC10528590 37429047

[25] Li HB, Dai JL, Wang ZX, Zheng HS, Li WL, Wang M and Cheng F. 2023. Digital light processing (DLP)-based (bio)printing strategies for tissue modeling and regeneration. Aggregate 4, e270.

[26] Gu ZM, Fu JZ, Lin H and He Y. 2020. Development of 3D bioprinting: from printing methods to biomedical applications. Asian J. Pharm. Sci 15, 529–557. 33193859 10.1016/j.ajps.2019.11.003 PMC7610207

[27] Li P, Zhang M, Chen ZY, Tian B and Kang X. 2023. Tissue-engineered injectable gelatin–methacryloyl hydrogel-based adjunctive therapy for intervertebral disc degeneration. ACS Omega 8, 13509–13518. 37091429 10.1021/acsomega.3c00211 PMC10116505

[28] Desai SU, Srinivasan SS, Kumbar SG and Moss IL. 2024. Hydrogel-based strategies for intervertebral disc regeneration: advances, challenges and clinical prospects. Gels 10, 62. 38247785 10.3390/gels10010062 PMC10815657

[29] Wang Y, Xu YD, Shang LJ and Mao YJ. 2023. GelMA hydrogel scaffold containing curcumin-loaded solid lipid nanoparticles promotes the regeneration of degenerative discs. SN Appl. Sci 5, 243.

[30] Barcellona MN, McDonnell EE, Samuel S and Buckley CT. 2022. Rat tail models for the assessment of injectable nucleus pulposus regeneration strategies. JOR Spine 5, e1216. 36203865 10.1002/jsp2.1216 PMC9520766

[31] Paesold G, Nerlich AG and Boos N. 2007. Biological treatment strategies for disc degeneration: potentials and shortcomings. Eur. Spine J 16, 447–468. 16983559 10.1007/s00586-006-0220-y PMC2229827

[32] Kim KW, Chung HN, Ha KY, Lee JS and Kim YY. 2009. Senescence mechanisms of nucleus pulposus chondrocytes in human intervertebral discs. Spine J. 9, 658–666. 19540815 10.1016/j.spinee.2009.04.018

[33] Roberts S, Evans H, Trivedi J and Menage J. 2006. Histology and pathology of the human intervertebral disc. J. Bone Joint Surg. Am 88 Suppl 2, 10–14. 10.2106/JBJS.F.00019 16595436

[34] Takeoka Y 2020. Reduced nucleotomy-induced intervertebral disc disruption through spontaneous spheroid formation by the Low Adhesive Scaffold Collagen (LASCol). Biomaterials 235, 119781. 10.1016/j.biomaterials.2020.119781 31981764

[35] Yue K, Trujillo-de Santiago G, Alvarez MM, Tamayol A, Annabi N and Khademhosseini A. 2015. Synthesis, properties, and biomedical applications of gelatin methacryloyl (GelMA) hydrogels. Biomaterials 73, 254–271. 26414409 10.1016/j.biomaterials.2015.08.045 PMC4610009

[36] Nichol JW, Koshy ST, Bae H, Hwang CM, Yamanlar S and Khademhosseini A. 2010. Cell-laden microengineered gelatin methacrylate hydrogels. Biomaterials 31, 5536–5544. 20417964 10.1016/j.biomaterials.2010.03.064 PMC2878615

[37] Gebhard H, James AR, Bowles RD, Dyke JP, Saleh T, Doty SP, Bonassar LJ and Härtl R. 2011. Biological intervertebral disc replacement: an in vivo model and comparison of two surgical techniques to approach the rat caudal disc. Evid. Based Spine Care J 2, 29–35. 22956934 10.1055/s-0030-1267084 PMC3427968

[38] Ishiguro H 2019. Intervertebral disc regeneration with an adipose mesenchymal stem cell-derived tissue-engineered construct in a rat nucleotomy model. Acta Biomater. 87, 118–129. 30690206 10.1016/j.actbio.2019.01.050

[39] Oswald KAC, Bigdon SF, Croft AS, Bermudez-Lekerika P, Bergadano A, Gantenbein B and Albers CE. 2021. Establishment of a novel method for spinal discectomy surgery in elderly rats in an in vivo spinal fusion model. Methods Protoc. 4, 79. 34842793 10.3390/mps4040079 PMC8628999

[40] Hall M, Frank E, Holmes G, Pfahringer B, Reutemann P and Witten IH. 2009. The WEKA data mining software: an update. ACM SIGKDD Explor. Newsl 11, 10–18.

[41] Arganda-Carreras I, Kaynig V, Rueden C, Eliceiri KW, Schindelin J, Cardona A and Sebastian Seung H. 2017. Trainable Weka Segmentation: a machine learning tool for microscopy pixel classification. Bioinformatics 33, 2424–2426. 28369169 10.1093/bioinformatics/btx180

[42] Polan DF, Brady SL and Kaufman RA. 2016. Tissue segmentation of computed tomography images using a Random Forest algorithm: a feasibility study. Phys. Med. Biol 61, 6553–6569. 27530679 10.1088/0031-9155/61/17/6553 PMC5039942

[43] Mitra I, Bose S, Dernell WS, Dasgupta N, Eckstrand C, Herrick J, Yaszemski MJ, Goodman SB and Bandyopadhyay A. 2021. 3D Printing in alloy design to improve biocompatibility in metallic implants. Mater. Today 45, 20–34. 10.1016/j.mattod.2020.11.021 PMC8248902 34220288

[44] Le Maitre CL, Dahia CL, Giers M, Illien‐Junger S, Cicione C, Samartzis D, Vadala G, Fields A and Lotz J. 2021. Development of a standardized histopathology scoring system for human intervertebral disc degeneration: an Orthopaedic Research Society Spine Section Initiative. JOR Spine 4, e1167. 34337340 10.1002/jsp2.1167 PMC8313169

