# Supplementary material for: 3D Bioprinted Chondrogenic Gelatin Methacrylate-Poly(ethylene glycol) Diacrylate Composite Scaffolds for Intervertebral Disc Restoration
Source: Int J Extrem Manuf. Author manuscript; Available in PMC 2025 Aug 26. (PMC12377657; doi:10.1088/2631-7990/ad878e)
Supplement: 1 [file NIHMS2034619-supplement-1.pdf]

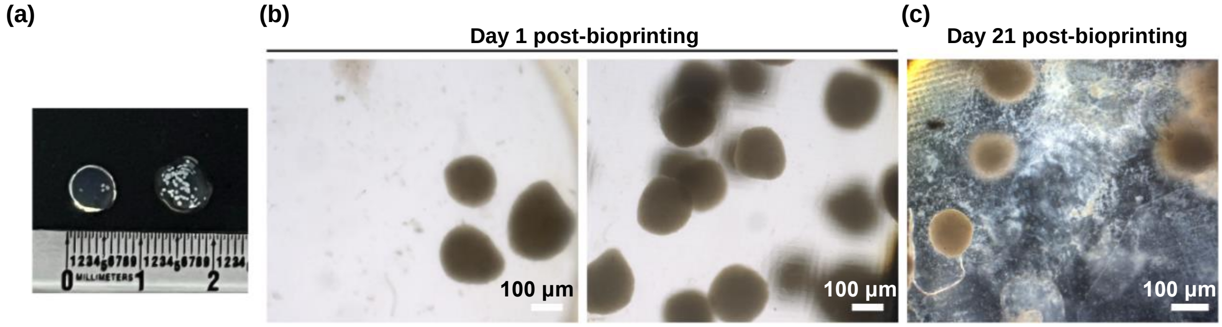

Figure S1. Spheroid density variation post-bioprinting. (a) Comparative images of GP10 scaffolds post-bioprinting with low spheroid density (left, approximately 4 spheroids) and high spheroid density (right, more than 20 spheroids). (b) Light microscopic images of hydrogel scaffolds containing low vs. high spheroid densities on day 1 post-bioprinting, providing an initial view of spheroid integration and scaffold structure. (c) Representative long-term maintenance and structural integrity of high-density spheroids within the scaffolds on day 21 post-bioprinting.

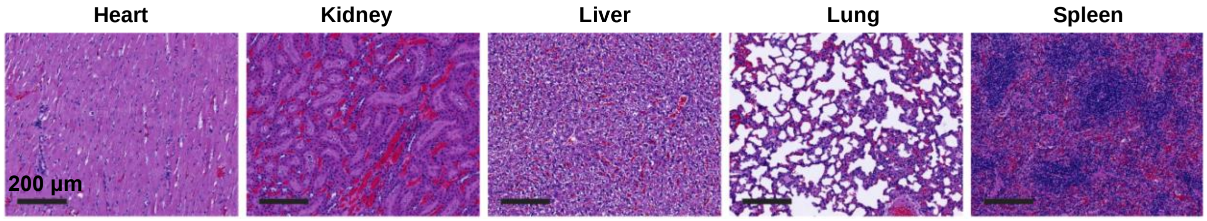

Figure S2. Representative H&E staining of major organs in rats 4 weeks after implantation with GP10 and rBMSC spheroids-GP10 hydrogel scaffolds revealing no evidence of toxicity or adverse tissue reactions, affirming the biocompatibility of hydrogel material, and confirming that degradation fragments of implanted scaffolds either remain localized or, if they enter circulation, do not accumulate in organs to elicit a systematic response.

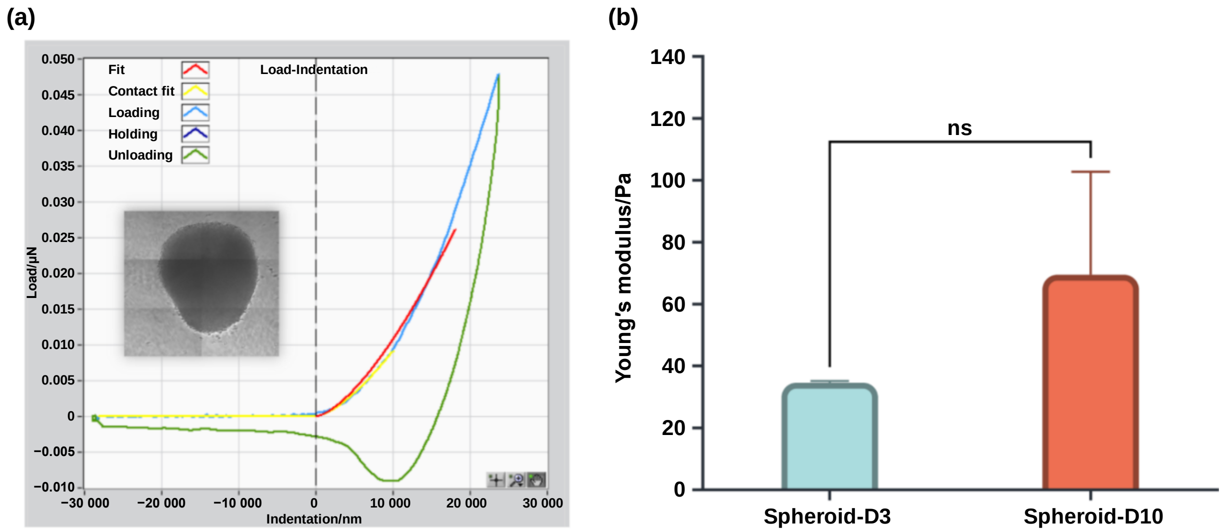

Figure S3. Mechanical characterization of spheroids at early culture stages. (a) Load-indentation curve for a representative spheroid, illustrating its mechanical response under compression. (b) Young's modulus (measured in Pascals) for spheroids cultures for 3 days and 10 days, indicating mechanical stiffness at these early time points. Ns= non-significant findings with p-value set at 0.05.
